# Microbial keratitis in Gujarat, Western India: findings from 200 cases

**Published:** 2011-11-29

**Authors:** Anil Kumar, Snehal Pandya, Ghanshyam Kavathia, Sejul Antala, Molly Madan, Tanuja Javdekar

**Affiliations:** 1 Department of Microbiology, Amrita Institute of Medical Sciences, Ponekara, Kochi-682041, Kerala India; 2 G.T.Seth Eye Hospital, Rajkot, Gujarat, India; 3 P.D.U. Medical College, Civil Hospital Campus, Rajkot, Gujarat, India; 4 Department of Microbiology, Subrati Medical College, Merrut, Uttar Pradesh, India; 5 Department of Microbiology, Baroda Medical College, Baroda, Gujarat, India

**Keywords:** Microbial, keratitis, village healer, eyes, epidemiology, India

## Abstract

**Introduction:**

The objective of this study was to study the epidemiological characteristics and the microbiological profile of patients suspected with microbial keratitis in Gujarat.

**Methods:**

Corneal scraping was collected from 200 consecutive cases of suspected microbial keratitis and was subjected to direct examination and culture.

**Results:**

Of the 200 ulcers 55% were culture positive, 26.5% were bacterial ulcers of which 47% were due to Staphylococcus spp. Pure fungal growth was seen in 22% while 6% were mixed ulcers. *Fusarium* spp. (30%) was the most common fungus followed by *Aspergillus* spp. (21%). Only one case of *Acanthamoeba* keratitis was encountered. Patients were mainly from rural areas (61.5%) with male preponderance (61.5%). Corneal injury was seen in 78.5% cases of which 53% had injury with vegetative matter. Prior treatment was seen in 58% of which 5% had been treated by village healers. Nineteen patients (9.5%) also used some kind of traditional topical treatment. Increased incidence was seen from August to December. Five case of fugal ulcers lead to perforation of which three were due to *Fusarium* spp. whereas perforation was seen in only two cases of bacterial ulcers due to *Pseudomonas aeruginosa*.

**Conclusion:**

*Staphylococcus* and *Fusarium* spp. were the most common etiological agents in our region. Predominant outdoor agricultural activity is the principal causative factor for corneal injury. Corneal ulcers complicated due to treatment by village healers are another important concern. The information regarding regional etiology will help empirical management as many eye clinics do not have microbiological facilities.

## Introduction

Infectious keratitis has for long been the Achilles’ heel of most ophthalmic surgeons. Corneal ulceration is a major cause of monocular blindness in developing countries. Ocular trauma and corneal ulcers annually results in 1.2 to 2 million cases of corneal blindness globally with 90% of them occurring in developing countries [1]. Corneal lesions were found to be responsible for 9% of all blindness in our country in a recent national survey by government of India [2]. The incidence of corneal ulcer was found to be as high as 1130 per million in a population based survey in south India [3]. Successful treatment of this condition depends upon accurate and rapid identification of the causative organism [1].

The epidemiology of causative agent's in microbial keratitis varies significantly from country to country and even from region to region within the same country. To devise a comprehensive strategy for diagnosis and treatment of corneal ulcers it becomes very important to determine the regional etiology within a given region. Several Indian studies have generated data to address this question but only one of them on mycotic keratitis has been published from the state of Gujarat [4]. Untreated infectious keratitis may result in corneal perforation, with the potential for development of endophthalmitis and the loss of the eye. Infectious keratitis can occur in any part of the cornea, but the infection involving the central cornea is of paramount importance. Scarring in this location has the potential to cause visual loss, even if the infecting agent is successfully eradicated, while some bacteria(e.g. *Gonococcus*) can invade intact epithelium, most infectious keratitis develop at the site of an abnormality or defect in the corneal surface [5].

This study describes the etiopathogenesis of microbial keratitis seen in a semi urban large government eye hospital in Gujarat over a period of two years September 2003 to August 2005. We also tried to determine the risk factors predisposing for the development of microbial keratitis with the objective of helping the clinicians in better management of such cases in this region.

## Methods

Two hundred consecutive patients with infectious corneal ulcers presenting to the ophthalmology department from September 2003 to June 2005 were included in the study. Ethics committee approval was obtained to conduct the study. Our hospital is a referral center that provides free eye care for patients from all Saurashtra and Kutch regions. Patients were enrolled after obtaining informed consent, the initial clinical diagnosis of corneal ulceration was made. Ulceration was defined as a loss of the corneal epithelium with underlying stromal infiltration and suppuration associated with signs of inflammation with or without hypopyon. Typical viral ulcers and healing ulcers were excluded as were Mooren's ulcers, marginal ulcers, interstitial keratitis, sterile neurotrophic ulcers, and any ulcers associated with autoimmune conditions. A standardized form was filled out on each patient documenting sociodemographic information as well as clinical information including duration of symptoms, previous treatment, predisposing ocular conditions, and associated risk factors.

### Clinical Procedures

All patients were examined under a slit-lamp biomicroscope by an ophthalmologist. Corneal scrapings were collected after instillation of 4% lignocaine without preservative under aseptic conditions from each ulcer by an ophthalmologist using a sterile Bard Parker blade (No 15) [6]. Scrapings were performed under magnification of slit-lamp or operating microscope. Leading edge and base of each ulcer were scraped initially and the material obtained were directly inoculated onto the surface of solid media such as sheep blood agar, chocolate agar and Sabouraud dextrose agar (SDA) in a row of C- shaped streaks and also deep inoculation in the liquid media such as brain heart infusion (BHI) broth without gentamicin sulphate and thioglycollate medium. Subsequent scrapings were spread onto labelled slides in a thin, even manner for 10 % potassium hydroxide (KOH) wet mount and Gram staining. Kinyoun's method of acid fast staining was performed only in cases of suspected actinomycetes keratitis. In cases of suspected Acanthamoeba keratitis the materials were inoculated onto non-nutrient agar overlaid with heat killed *Escherichia coli* broth culture. Strict asepsis was maintained in the collection and transferring of scraped material it to the appropriate culture media.

### Laboratory procedures

All bacterial cultures were incubated aerobically. Cultures on blood agar and chocolate agar were evaluated at 24 hours and at 48 hours and then discarded if no growth was seen. Chocolate agar was incubated in a candle jar, to provide 5 % CO_2_, when Pneumococci were suspected. All media's were incubated at 35°C (±1) except SDA, which are incubated at 27°C (±1) in BOD incubator. Petri dishes were incubated with lids facing downwards to prevent condensed moisture from dripping on to the medium. Cultures inoculated in BHI broth were examined for turbidity in similar fashion which was subsequently subcultured, and Gram stained for identification. However liquid media were prone to contamination and were not used for interpretation in isolation. The criteria described by Bharathi et al. were used for determining culture positive samples [6].

Cultures for *Staphylococcus epidermidis* and *Diphtheroids* were considered positive only if there was moderate growth on at least two solid media. The specific identification of bacterial pathogens was based on microscopic morphology, staining characteristics, and biochemical properties using standard laboratory criteria. Fungi were identified by their colony characteristics on SDA and by their microscopic appearance in Lactophenol cotton blue. Cultures on non-nutrient agar (NNA) overlaid with E coli were examined daily for the presence of *Acanthamoeba* spp. and likewise discarded at one week if there were no signs of growth. After identification of the organism, antibiotic susceptibility testing of each isolate was done according to disc diffusion technique by Kirby Bauer method on Muller Hinton agar (MHA) for non-fastidious organisms and on MHA with sheep blood for *Pneumococci*.

## Results

### Epidemiological characteristics

Out of 200 patients 122(61%) were males and 78(39%) were females. There were 123(61.5%) rural residents and 87(38.5%) urban residents. Patients above the age of 50 years (71, 35.5%) were significantly less than patients below 50 years (129,64.5%). Non-agricultural workers (Table 1) were significantly less in number than were farmers (108, 54%). Co-existing ocular diseases predisposing to corneal ulceration were identified in 24 (12%) patients, compared to other predisposing risk factors in 177 (88.5%) patients. A history of corneal injury was recorded in 157 (78.5%) patients, of which 83 (53%) had corneal injury with vegetative matter and 12 (7.6%) had injury due to fingernail (Table 2). Of 200 patients, 108 (54%) patients had corneal ulcers in the right eye, 92 (46%) in the left eye and 1 (0.5%) in both eyes. The seasonal distribution of 200 culture positive bacterial keratitis cases analyzed over a period of two years. Our data revealed that in western India, there was increased incidence of microbial keratitis from September to December than other months. The surprising finding was that out of the 200 cases 10 (5%) had taken treatment from quacks/village healers to remove foreign body from their eyes. Prior treatment with topical medication was noted in 116 (58%) of the patients of which 72 (62.06%) were on topical antibiotics, 15 (12.93%) were on antifungals, and 29 (25%) were on corticosteroids. It was of interest that 19 patients (9.5%) were also using some kind of traditional or herbal topical treatment.

**Table 1:** Occupation of patients with corneal ulceration (n=200) in a tertiary care institution in Gujarat, India

Occupation	Number	Percentage %
Agriculture worker/farmer	108	54%
Housewife/domestic	20	10%
Laborer*	22	11%
Tradesman/Profession†	14	7%
Student/Child	12	6%
Unemployed/Unknown	24	12%
Total	200	100%

* An individual either male or female, who does heavy manual labor, lifting loading & carrying of material usually balanced on head;

† Middle class workers such as mechanics, stonemasons, electricians, carpenters, pipe fitters, welders, and fishermen. This category also includes profession as teachers, police, office workers, factory workers, drivers and merchants

**Table 2:** Traumatic agents in patients with corneal ulcerations (n=157) in a tertiary care institution in Gujarat, India

Traumatic agents	Cases	%
Tree branch or thorn	20	12.7
Dust, soil or stones	18	11.5
Vegetable matter^a^	63	40.12
Animal matter^c^	3	1.9
Metallic foreign body	4	2.5
Miscellaneous^b^ objects	3	1.9
Finger nail	12	7.6
Contact lens	1	0.6
Unknown	33	21
Total	157	100

^a^ Organic matter, hay, sugarcane, grass, corn stalks, wood, onions, groundnuts, palm leaf

^b^ Metallic objects, chemicals, paint, fishnets, broomsticks, cricket balls, cloth, etc

^c^ Cow tail, cow dung

### Microbiological diagnosis

Cultures were positive and fulfilled the criteria established for the presence of infection in 110 (55%) of the 200 corneal ulcers (Table 3). Pure bacterial growth was present in 53 (26.5%) of the 200 cultures performed and pure fungal growth in 45 (22.5%). Mixed microbial growth was present in the cultures of 12 (6%) of the 200 patients and there was one case of Acanthamoeba keratitis (0.5%). A total of 70 bacterial organisms were cultured from 65 corneal ulcers (Table 4). Of the 70 isolates, 44 (72.86%) were Gram positive and 14 (27.15%) were Gram-negative bacteria. *Staphylococcus* spp. was the most commonly isolated bacterial organism representing 33 (47.4%) of all positive bacterial cultures. The next most commonly isolated Gram-positive organism was *Streptococcus pneumonia* with 9 (12.86%) positive cultures. Of these 9 cultures, six were pure, one was mixed with other bacteria, and two were mixed with fungi. Only one culture was positive for *Nocardia asteroids* (1.42%) and was also included under Gram-positive bacterial species. *Pseudomonas aeruginosa* was isolated from 12 cultures (17.14%) and was the most frequently occurring Gram-negative organism. A total of 57 fungal organisms were cultured from equal number of corneal ulcers (Table 5) of which 17 (29.82%) were *Fusarium* spp., 12 (21.05%) were *Aspergillus* spp., and 8 (14%) were demateacious fungi. Eight other fungal species were cultured in decreasing frequency along with 9 (15.79%) unidentified hyaline fungal species. Of the 57 fungal ulcers 39 (68.4%) were positive for fungal elements on KOH examination. One case of an HIV positive patient with corneal ulcer due to *Moraxella* spp. was seen. We had also encountered one case of pigmented plaque keratitis due to *Curvularia* spp. following thorn injury. There was also a case of post cataract mixed corneal ulcer due to *Aspergillus* spp. and *Pseudomonas aeruginosa* as a result of corticosteroids abuse. The most common home remedy was the application of breast milk into the eye, although patient applied various other materials as well. Of all the fungal ulcers five cases lead to perforation in spite of treatment and had to undergo therapeutic tectonic keratoplasty with cryopreserved cornea. Out of them three cases were due to *Fusarium* spp., one due to *Aspergillus flavus* and one remained unidentified hyaline fungus. Only two cases of pure bacterial ulcers led to perforation and both were due to *Pseudomonas aeruginosa*. The most common bacterial isolate Staphylococcus was found highly sensitive to Cefazolin (94%) while ceftazidime was found to be most effective for *Pseudomonas aeruginosa*. Not a single case of methicillin resistant *Staphylococcus aureus* was encountered.

**Table 3:** Microbial growth pattern in cultures from corneal ulcers (n=200) in a tertiary care institution in Gujarat, India

Growth pattern	Cases	%
Pure bacterial growth (Single species of bacteria)	48	24
(Two species of bacteria)	5	2.5
Pure fungal growth (Single species of fungus)	45	22.5
Mixed microbial growth (Single species of bacteria with a single species of fungi)	12	6
Pure *Acanthamoeba* ulcer	1	0.5
Patients with positive cultures	110	55
Patients with negative cultures	90	45
Total number of ulcers	200	100

**Table 4:** Bacterial isolates from corneal ulcers in a tertiary care institution in Gujarat, India

Bacteria	Pure isolate	Mixed with fungi	Total	%
**Gram positive**	**7**	**2**	**9**	**12.86**
*Streptococcus pneumoniae*	6	1	7	10
*Diptheroids spp*.				
*Staphylococcus epidermidis*	9	3	12	17.14
*Staphylococcus aureus*	20	1	21	30
*Streptococcus pyogens*	1	0	1	1.43
*Nocardia spp*.	1	0	1	1.43
Sub total	44	7	51	72.86
				
**Gram negative**				
*Pseudomonas aeruginosa*	7	5	12	17.14
*Moraxella spp*.	2	0	2	2.86
*Proteus mirabilis*	1	0	1	1.43
*E. coli*	3	0	3	4.29
*Klebsiella pneumoniae*	1	0	1	1.43
Sub total	14	5	19	27.15
Total	58	12	70	100

**Table 5:** Fungal isolates from corneal ulcers in a tertiary care institution in Gujarat, India

Fungi	Pure isolates	Mixed with bacteria	Total	%
*Fusarium* spp.	14	3	17	29.82
*Aspergillus* spp.	9	3	12	21.05
*Curvularia* spp.	4	1	5	8.77
*Alternaria* spp.	2	–	2	3.50
*Bipolaris* spp.	1	–	1	1.75
*Aurebasidium pullolans*	1	–	1	1.75
*Scorpulopsis* spp.	1	–	1	1.75
*Colletotricum* spp.	1	–	1	1.75
*Penicillium* spp.	2	1	3	5.26
*Paecilomyces* spp.	2	–	2	3.50
*Syncephalastrum* spp.	3	–	3	5.26
*Unidentified hyaline fungus*	5	4	9	15.78
Total	41	12	57	100

## Discussion

Microorganisms were isolated from 55% of the 200 cases of presumed microbial keratitis which is close to many other reports [7,8] but it does not approach the high isolation rates reported from Nepal [9]and Bangladesh [10] even though multiple scraping were performed and enriched media was used for inoculation. The low rates of isolation were attributed to the more widespread availability of topical medications as reported by Srinivasan et al. [8].

Monomicrobial infection was seen in majority (84.5%) of the cases the most common being bacterial (48%). Similar figures have been reported from Madurai [7], Tirucharapalli [10], and south Ghana [7,11]. In mark contrast high prevalence of bacterial keratitis was reported from Hyderabad [12] and Nepal [9]. *Staphylococcus* spp. 33(47.4%) was the predominant bacterial species in this study and was similar to the reports from prior Indian studies [12,13]. and other parts of the world [5]. The lone study done in this region (unpublished data) also implicated *Staphylococcus* spp. to be the most common (56.30%). In contrast, predominance of *Pseudomonas aeruginosa* in Bahrain [14], Ghana [6] and Hong Kong [15] and *Streptococcus pneumonia* was predominant bacterial species in the reports from Madurai [8], Trichirapalli [11] and Nepal [9] and Tirunelveli [16]. These reports show that there is distinct pattern of geographical variation in the aetiology.

The frequency of keratitis was greater in men than in women. The former were affected almost 1.5 times more than the latter (61% vs. 39%). This is in accord with prior studies [12,17,18] and from other parts of the world [5], where male preponderance has been established in a ratio ranging from 1.5:1 to 4.5:1 but in variance with the findings of Poria et al. [4] from this region, Al-Yousuf [14]. ,R.Maske et al. [18] and Subbannaya et al. [19] who found females to be more affected. By the nature of their work profile, men are more exposed to outdoor activities, thereby increasing their vulnerability to the disease. There was a significant higher incidence of bacterial /fungal keratitis among patients who were agricultural workers (54%) This study shows that there is significant association between occupation and microbial keratitis.

Microbial keratitis is significantly higher (73.62%) among those aged < 50 years in our present study. Corneal trauma is the leading cause of microbial keratitis [8,10,15,20] which were also found in the present study (78.5%). In eastern India, hay or wheat/maize/groundnut stalks in the field was the most common cause of superficial corneal trauma. Twenty five per cent of all patients with a history of trauma implicated wheat/maize stalks as the traumatic object. This was followed by tree branches and thorns, soil and rocks, vegetable matter, animal products and metal objects. Another common risk factor, according to reports from developed countries, has been documented to be contact lens wear. This has been implicated in 6–29% of the infective keratitis cases in such nations. [14,21,22]. In our study only one (0.5%) case was found using contact lenses in accordance with studies from Chandighar [20,23]. ,Tirunelveli [16], Hyderabad [12] and Nepal, [9]. This low prevalence may be due to the fact that the majority of the patients were from the lower socioeconomic class.

Almost half (40.9%) of all corneal ulcers with positive cultures were fungal in origin. If the 12 mixed infections (10.9%) are also considered to be primarily fungal for treatment purposes and they are added to the pure fungal cases, 51.8% of all culture positive corneal ulcers grew fungal pathogens. This figure approaches the fungal isolation rate by Hagan et al. [7] (56%) and Srinivasan et al. [8] (51.9%).While bacterial keratitis was seen almost throughout the year the incidence of fungal keratitis was highest from August to December. There was a let down in February and then peaked in March and April because of wheat/maize/groundnut harvesting and other agricultural activities. This is substantiated by Panda et al. [24] and Gopinathan et al. [25]. Previous data report that fungal keratitis is most common in the sixth decade, i.e., 51–60 years [8,9].We, however, found that this disease involves the younger subgroup 20–40 years most frequently (41%). Because patients in the third to fourth decade age group are often the breadwinners of the family, the blindness is of much greater economic consequence. Only previous study by Sandhu et al. [26] and Gopinathan et al. [25] have agreed with our finding of fungal keratitis being most common in the third decade which is in variance with the findings of Sharma et al. [27] Poria et al. [4] and Subbannaya et al. [19] who found maximum prevalence between 41-60 years of age.

The prevalence of 22.5% of fungal keratitis in our case series is at variance with other large hospital- based series by Chander et al., [17] but in concordance with the figure of 32% quoted by Sandhu et al. [26] in a study from Amritsar. The corresponding figures from similar hospital-based data of South India are commonly 11– 47% [8,12,23].Of 57 fungal isolates cultured from equal number of corneal ulcers 29.82% were *Fusarium* spp., 21.05% were *Aspergillus* spp. and No non-filamentous fungi were cultured from any of the patients. This pattern of fungal organisms, dominated by *Fusarium* spp, is similar to the spectrum of microbial keratitis reported from South Florida [5] and from Ghana [7].The various Indian studies implicating *Fusarium* spp. as the predominant fungal species are from Bharathi et al. [16]; Garg et al. [28] & Srinivasan et al. [8]. The only published data on mycotic ulcers from this region by Poria et al. [4] also found *Fusarium* spp. to be the most common followed by *Aspergillus* spp.. By contrast, Aspergillus spp. was the most common fungus reported from Nepal [9], Bangladesh [10], West Bengal [13], parts of South India [28] and Mumbai [29].

It is of interest that over 40% of the patients in the study presented for examination during the first week of their illness and 28.5% reported in the second week is similar with findings in Nepal [10].Majority of patients in eastern India don't appear to have access to relatively sophisticated eye care. Before their initial examination 116(58%) patients consulted a healthcare provider of some kind in agreement with findings from west Bengal [13] and Hyderabad [12]. Of all patients seeking medical attention 8.6% [10] went to a village healer. Home remedy was used in 9.5% cases. Similar findings have been reported from Madurai (37.5%) [8] and Hyderabad (0.4%) [12]. The most common home remedy was the application of breast milk into the eye, although patients applied various other materials as well. Courtright et al. [30] described the use of traditional eye medicines among patients with corneal diseases in rural Malawi.

## Conclusion

Microbial keratitis continues to be an important cause of ocular morbidity and a cause for concern among ophthalmologist in Gujarat and it is evident that predominant outdoor agricultural activity and vegetative injury are the principal causative factor. The information regarding regional etiology is important with regard to empirical management of corneal ulcers as it will help in limiting ocular morbidity since many eye clinics do not have microbiological facilities. Corneal ulcers complicated due to treatment by village healers are another important concern and needs to be addressed by educating the rural population.
